# Molecular epidemiology and associated risk factors of rotavirus infection among children < 5 yrs hospitalized for acute gastroenteritis in North Eastern, Kenya, 2012

**DOI:** 10.11604/PAMJ.SUPP.2017.28.1.2486

**Published:** 2017-11-03

**Authors:** Ahmed Mohamed Fidhow, Amwayi Samwel, Zipporah Ng’ang’a, Joseph Oundo, James Nyangao, Arvelo Wences

**Affiliations:** 1 Field Epidemiology and Laboratory Training Program, Ministry of Public Health and Sanitation, Kenya; 2 Jomo Kenyatta University of Agriculture and Technology, Kenya; 3 United States Army Medical Research Unit, Kenya; 4 Kenya Medical Research Institute, Kenya; 5 Centers of Disease Control and Prevention, Kenya

**Keywords:** Rotavirus, hospitalized, acute gastroenteritis, RNA profiles, genotypes, risk factors, aetiology, rotavirus vaccines

## Abstract

**Introduction:**

Rotavirus is a leading cause of morbidity and mortality among children under five years worldwide. This study aimed to characterize the circulating genotypes of rotavirus and to determine risk factors of rotavirus infection in North Eastern, Kenya before the introduction of rotavirus vaccines.

**Methods:**

we conducted a cross sectional study among children < 5 years old hospitalized for acute gastroenteritis at the study hospital. Rotavirus was detected in stool specimens and further characterized using PAGE and RT-PCR. Socio-demographic and risk factor information was collected using a standard questionnaire.

**Results:**

we enrolled 237 children into the study hospitalized with acute gastroenteritis. Of these, 41 (17%) tested positive for group A rotavirus in stool specimens. Age < 2 years, unboiled tap water, underweight and low birth weight were identified as independent risk factors of rotavirus infection. Majority 8 (57%) of the detected rotavirus RNA profiles were long electropherotypes. G3, G9 and P4 were the predominant genotypes identified.

**Conclusion:**

Rotavirus is an important aetiology of acute gastroenteritis among children under five years in this region. Risk factors common in other regions and rotavirus vaccine preventable genotypes are responsible for infection. We recommend the introduction of rotavirus vaccines, coupled with good infant nutrition, safe water supply and maternal hygienic practices during infant feeding.

## Introduction

Diarrheal diseases remain the leading cause of mortality among children under 5 years around the world [1]. Each year, more than one billion episodes of diarrhoea occur among children under five years causing approximately 2.5 million deaths [2]. Among these Rotaviruses are globally the leading cause of severe, dehydrating diarrhea in children aged < 5 years [3]. Each year two million children younger than five years are hospitalized with rotavirus acute gastroenteritis and an estimated 527,000 children die [4]. In Kenya, rotavirus infection causes 19% of hospitalizations and 16% of clinic visits for diarrhea among children < 5 years of age, and results in 4471 deaths, 8781 hospitalizations, and 1,443,883 clinic visits yearly [5].

The enormous burden of rotavirus diseases has made the development of Rotavirus vaccines a global priority [6]. A vaccine to protect against rotavirus diarrhea, RotaShield (Wyeth Laboratories, Marietta, PA), was licensed in 1998 and shortly thereafter withdrawn because of an increased risk of intussusception [7]. Two rotavirus vaccines have now been licensed or are nearing licensure in many parts of the world; a live attenuated G1P [8] human RV vaccine (Rotarix; GlaxoSmithKline Biologicals, Rixensart, Belgium), licensed in Europe and more than 60 other countries around the world, and a pentavalent live human-bovine reassortant vaccine (Rotateq; Merck & Co., Whitehouse Station, NJ) licensed in the United States, Europe, and Australia [8]. Extensive phase III trials for these vaccines showed high efficacy in protecting children against rotavirus disease of any severity, for strains with the same serotypes as contained in the respective vaccine, and there was a significant degree of cross-reactivity against many genotypes not contained in the vaccines [9]. In 2006, WHO strongly recommended the inclusion of these new rotavirus vaccines into national immunization programs of countries in the Americas and Europe on the basis of pivotal clinical trials from these regions [10]. In 2009, WHO recommended the inclusion of rotavirus vaccination of infants into all national immunization programmes [11]. To assess the potential impact of these vaccines in sub-Saharan Africa, where rotavirus mortality is high, knowledge of prevalent types is essential because an effective rotavirus vaccine is needed to protect against prevailing serotypes in the community [12].

In Kenya, data on rotavirus strain prevalence and disease burden is only limited to few facilities in the urban settings and the few studies that were conducted mostly concentrated in urban areas. The objectives of this study were therefore to determine the risk factors associated with rotavirus infection and to describe the circulating genotypes of rotavirus among children under five years hospitalized for acute gastroenteritis in Garissa, Kenya before the introduction of rotavirus vaccines with an aim to recommend an appropriate prevention and control measures for rotavirus infection and to guide policy decision makers on the choice of vaccination strategy to implement.

## Methods

### Study site

We conducted the study in Garissa Provincial General Hospital which is situated in Garissa Central in Garissa County, which is one of the three Counties in North Eastern Province of Kenya. The hospital is the largest public hospital in North Eastern Province of Kenya and serves as a teaching and a referral hospital to several district health facilities in the region as well as to other neighbouring district hospitals from Eastern and Coastal Provinces of Kenya. Garissa County has a population size of 623,060 persons [13] and covers a land size of 44175 sq km [14].

### Study design

We conducted a cross sectional survey among children < 5 years old hospitalized for acute gastroenteritis at Garissa Provincial General Hospital in North Eastern Province of Kenya between February and June 2012. A case of acute gastroenteritis was defined as three or more episodes of loose stools of sudden onset with/out fever and/or vomiting. A sample size of 237 was obtained using a systematic random sampling technique. A standard questionnaire was used to collect socio-demographic, clinical and risk factor information from all children enrolled into the study.

### Collection and transportation of stool specimens

We attempted to collect 5mls of stool specimen from each participant in a clean, and detergent free container. Specimens were initially processed at the study hospital laboratory and then shipped to Nairobi for analysis in Kenya Medical Research Institute Rotavirus Laboratory. Storage of specimens at the study hospital laboratory was done at -200C and shipment to KEMRI Rotavirus laboratory was done at -20oC on dry ice.

### Group A Rotavirus (GARV) screening and molecular typing

Stool suspensions were made prior to the testing procedure and a commercially available enzyme-linked Immunosorbent assay ELISA kit (Prospect) was used to detect group A Rotavirus antigens in the stool specimens according to the manufacturer’s instructions. Rotavirus RNA electropherotypes in positive specimens were determined using polyacrylamide gel electrophoresis (PAGE) described by Herring *et al.* (1982) [15]. The VP7 specificity was genotyped using RT-PCR method described by Gouvea *et al.* (1990) [16] using a cocktail of VP7 specific primers to the six human serotypes (G1-G4, G8 and G9). The VP4 specificity was determined using RT-PCR method described by Gentsch et al .(1992) [17] similarly using a nested PCR reaction with a mixture of VP4 specific primers.

### Data analysis

We conducted data analysis using Epi Info version 3.5.1 and excel analysis software. Proportions and means were calculated for categorical and continuous variables respectively and summarized into tables and figures. Prevalence odds ratio was used to measure the significance of association between exposure variables and the outcome variable and chi-square test was used for significance testing with level of significance set at *p*<0.05.

### Ethics

The approval to conduct this study was granted by both the Kenya Medical Research Institute Scientific Steering Committee (KEMRI SSC) and the Kenya Medical Research Institute Ethical Review Committee (KEMRI ERC).

## Results

A total of 237 children with acute gastroenteritis were enrolled into the study. Females were more than the males 120 (51%). The mean age of the children was 23 months (SD+16 months) and the median age was 24 months (IQR = 8-36 months). Majority 50 (21%) were in the age-group 0-6 months, followed by age-group 7-12 months 39 (17%) and age-group 25-30 months 34 (14%). Table 1 shows the socio-demographic characteristics of children participating in the study.

**Table 1:** socio-demographic characteristics of children < 5 years hospitalized with acute gastroenteritis in North Eastern, Kenya

Variables	Frequency(N=237)	%
**Age-group (Months)**		
0-6	50	21
7-12	39	17
13-18	16	7
19-24	29	12
25-30	34	14
31-36	29	12
37-42	17	7
43-48	12	5
> 49	11	5
**Sex**		
Female	120	51
Male	117	49
**Maternal education level**		
No formal education	64	27
Primary level	114	48
Secondary level	44	19
Tertiary level	15	6
**Maternal occupation**		
Housewife	213	90
Informal	19	8
Formal	5	2

**Table 2:** bivariate analysis of socio-behavioral risk factors of rotavirus infection among children < 5 years hospitalized with acute gastroenteritis North Eastern, Kenya

Variables	Positive N (%)	Negative N (%)	cOR (95% C.I)	P value
Having age less than two years	29 (22)	105(78)	2.09 (1.01-4.34)	0.04
Being of female gender	23 (19)	97 (81)	1.30 (0.66-2.57)	0.44
History of exclusive breastfeeding	2 (4)	46 (96)	0.17 (0.04-0.72)	0.003
History of drinking of unboiled tap water	28 (30)	65 (70)	4.34 (2.11-8.94)	0.00003
Guardian hand hygienic practices	14 (11)	116 (89)	0.36 (0.18-0.72)	0.003
Being underweight	15 (39.5)	23 (60.5)	4.34 (2.0-9.37)	0.00008
Having history of low-birth weight	7 (50)	7 (50)	5.56 (1.83-16.86)	0.0009
Born to a mother without formal education	39 (22)	139 (78)	7.99 (1.94 -70.21)	0.003
Born to unemployed mother	38 (18)	175 (82)	1.52 (0.43-5.36)	0.37

**Figure 1:**
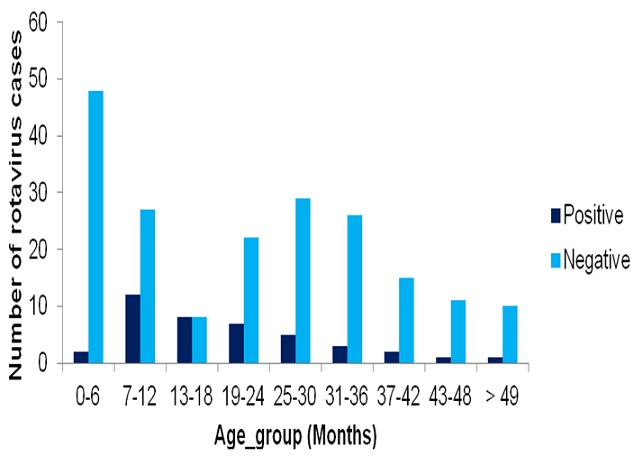
a map of Murchison-Semliki landscape showing location of Conservation & Ecosystem Health Alliance (CEHA) project activities

The overall prevalence of rotavirus infection among the children was 41 (17%), with the mean age of infection being 19 months (SD+13 months) and the median age being 18 months (IQR = 10-26). Majority of the infected children were below the age of two years, with the highest incidence of infection 12 (31%) occurring among children in the age-group 7-12 months, followed by age-group 13-18 months 4 (25%) and age-group19-24 months 7 (24%). Figure 1 shows the distribution of rotavirus cases among the study participants by age-group. Females were more infected 23 (19%) than the males 18 (15%), with male to female ratio of 1:1.2.

On bivariate analysis, children below the age of two years {OR = 2.09, CI (1.01-4.34), (*P =* 0.04)}, those that had consumed unboiled tap water {OR = 4.34, CI (2.11-8.94), (*P =* 0.00003)} and those with malnutrition {OR = 4.34, CI (2.01 9.37), (*P =* 0.00008)} were more likely to have rotavirus infection. Similarly, those born to mothers without formal education {OR = 6.67, CI (1.55-28.66), (*P =* 0.002)} and those with history of low birth weight {OR = 5.56, CI (1.83-16.86), (P = 0.0009)} were also more likely to have rotavirus infection. In contrast to the above, children that were exclusively breastfed {OR = 0.17, CI (0.04-0.72), *P =* 0.003)} and those with mothers practicing hand hygiene during child feeding {OR = 0.36 CI (0.18-0.72), (*P =* 0.003)} were less likely to have rotavirus infection. Maternal occupation and gender of the participants were not significantly associated with rotavirus infection. Table 2 shows bivariate analysis of socio-behavioral risk factors associated with rotavirus infection.

In multivariate analysis, all factors significant in bivariate analysis were identified as independent risk factors of rotavirus, except maternal education level and exclusive breastfeeding practices which were not significant. In contrast, hand hygiene among the mother’s of the children was identified as a protective factor against rotavirus infection. Table 3 shows independent risk factors of rotavirus infection.

**Table 3:** independent risk factors of rotavirus infection among children < 5 years hospitalized with acute gastroenteritis in North Eastern, Kenya

Variables	PositiveN (%)	NegativeN (%)	AOR (95% CI)	P value
Having age less than two years	29 (22)	105(78)	7.78 (2.76-21.93)	0.0001
History of exclusive breastfeeding	2 (4)	46 (96)	0.19 (0.04-1.02)	0.05
History of drinking unboiled tap water	28 (30)	65 (70)	4.65 (1.78-12.13)	0.002
Guardian hand hygienic practices	14 (11)	116 (89)	0.33 (0.14-0.79)	0.01
Being underweight	15 (39.5)	23 (60.5)	6.73 (2.35-19.27)	0.0004
History of low-birth weight	7 (50)	7 (50)	6.27 (1.32-29.71)	0.02
Born to a mother without formal education	39 (22)	139 (78)	15.72 (2.94-84.03)	0.001

On phenotypic analysis, only 14 (34%) of the rotavirus stool specimens showed distinct RNA profiles, comprising of long and short electropherotypes. Long electropherotypes comprised of 8 (57%) and short electropherotypes comprised of 6(43%). On further genotype analysis, a total of 3 G types and 4 P types of rotavirus were identified. Among the G types, G3 and G9 were both predominant in equal proportions of 41% (n = 7), followed by G1 with a prevalence of 4(24%). G2 and G4 were not detected in this study. Among the P types, P4 was predominant 7(44%), followed by P [6] and P [8] in equal proportions of 2 (13%). A substantial proportion of P-types were not type able P [NT] 5 (31%). A total of five G-P genotype combinations were detected in this study. Of these, G3P [4] was found to be predominant 7 (41%), followed by G9P [NT] 6(35%) and G1P [6] 2 (12%); other G-P genotypes such as G1P [8] and G9P [8] occurred as the least frequent genotypes with an equal prevalence of 6% (n = 1).

## Discussion

In this study, Rotavirus is an important cause of acute gastroenteritis among children under five years hospitalized for diarrhoeal disease occurring with a prevalence of 17%. Similar prevalence of rotavirus was reported in a study conducted in Maua Kenya (17.8%) [18]. However, a slightly lower prevalence of rotavirus was reported in studies conducted in Nigeria (13.8%) and Jamaica (15.0%) [19]. The reason for the difference in the prevalence rates of rotavirus infection may be explained by the different conditions of the studies, such as the period of sampling and the sampling technique used.

Previous studies around the world have identified several risk factors of rotavirus infection. In this study, having the age below two years, drinking of unboiled tap water, having any form of malnutrition, having history of low birth weight and being born to a mother who is not educated were all identified as independent risk factors of rotavirus infection. On the other hand, hand hygienic practices among the mothers of the respondents during child feeding were identified as a protective factor against rotavirus infection. In Tanzania, contrary to the findings of this study, rotavirus infection rates were found not to be significant between those who had malnutrition and those with normal nutrition [20]. However, according to Black et al, children who are small because of young age and/or malnutrition lost a greater proportion of their total body fluid during diarrhea and speculated that they may be expected to have a higher frequency of severe dehydration and death [21]. In Jordan, similar to the findings of this study, infant feeding practices of using unboiled tap water to prepare the formula milk, and the low educational level of the mothers were found to be risk factors of rotavirus infection [22].

Rotavirus has been described as a causative agent in several waterborne outbreaks in the industrialized countries indicating good survival of rotavirus in water [23]. In the US, similar to the findings of this study, Low-birth-weight (< 2500 g) infants were found to be at increased risk for hospitalization due to rotavirus even beyond the first few months of life [24]. Among the respondents investigated in this study, children below the age of two years had shown the highest burden of Rotavirus infection, with lower rates of infection occurring among children below the age of six months and a declining rate of infection observed among children older than two years. Similar findings were reported by studies conducted in Burkina Faso [25] and Nigeria [26]. The difference in the rates of Rotavirus infection between the agegroups can be explained by the protective effect of maternal antibodies in < 6 months old, and the development of natural immunity after repeated infections in children over 2 years of age. The high burden of rotavirus infection in young children highlights the need for vaccine to offer optimal protection against severe rotavirus disease in children aged younger than 2 years [27]. Gender distribution of Rotavirus was found not to be significant in this study, indicating that Rotavirus did not have a predilection for any gender category.

Epidemiologic studies of rotavirus infections are increasingly showing that a great diversity of rotavirus strains are cocirculating in the human population throughout the world [28]. The prevalence of rotavirus genotypes varies according to location and time. Throughout the world, genotyping and serotyping studies have identified common cocirculating rotavirus types, and G1P [8], G2P [4], G3P [8], and G4P [8] are the predominant strains. However, from time to time, other less common genotypes, such as G9P [8], G5P [8], and G8P [6], have been predominant in various countries.

In this study, both long and short electropherotypes were found in this region with long electropherotypes being predominant over the short electropherotypes. Similar finding was reported by a study conducted in Maua, Kenya, where 80% of the rotavirus RNA profiles that were detected comprised of the long electropherotype family [18]. Elsewhere in Africa, a study conducted in Cameroon also reported the predominace of long electropherotypes over short electropherotypes [29]. However in India, findings were contrary to this study, with short RNA electropherotypes being predominant over long electropherotypes [30].

Regarding the circulating genotypes of rotavirus, both G3 and G9 occurred in equal proportions as the predominant G-types followed by G1, while P4 occurred as the predominant P-type followed by P6 and P8 in equal proportions. The implication this has on rotavirus vaccine is that, both the current rotavirus vaccines have the potential to protect against the circulating genotypes in this region.

In a study that reviewed papers published over the last 30 years on the epidemiology of Rotavirus diarrhoea among the hospitalized and out-patient children in Kenya, G1 was found to be the most predominant up to the year 2002 [31]. In Kilifi Kenya, P [8] G1 (42%) was found to be the predominant strain, followed by P [8] G9 (15%) [32]. Elsewhere in Kenya, studies conducted in Nairobi, Nanyuki and Kitui, showed serotype G4 as the predominant rotavirus strain different from the findings of this study [33]. In Africa, findings from a study conducted in Morocco showed a similar distribution of rotavirus genotypes to the study findings, though the two studies differed in the frequency at which the genotypes occurred [34]. Of note from this study is the absence of G2 and G4 which are part of the globally important strains of rotavirus. Also animal strains have not been detected in this region despite the high background of human and animal interactions.

The high prevalence of P non-typeable strains in this study was presumably as a result of antigenic drift, and provides further evidence for extensive natural variation in rotavirus strains in this location. The variability in the genotype profiles has been shown in previous studies to be dependent on a given place and a period of time [35].

## Conclusion

In conclusion, rotavirus is an important aetiology of acute gastroenteritis among children under five years in this region. Current rotavirus vaccine preventable genotypes were found to be responsible for rotavirus transmission, with risk factors common in other regions also associated with infections in this region. We therefore consider the introduction of current rotavirus vaccines to children below the age of two years, coupled with good infant nutrition, safe water supply and public health education to mothers on good personal hygiene. We also recommend the continuous monitoring of rotavirus disease burden and strain distribution which might impact on the effectiveness of rotavirus vaccines after introduction.

Like many studies that were conducted in the past, this study had several limitations. The magnitude of rotavirus infection among the out-patient cases could not be established as the study focused on hospitalized cases only. Hospitalized cases were considered as a proxy to severe cases of rotavirus gastroenteritis. Being a cross sectional study, data comparing rates of rotavirus infection during the same season in different years were not available, hence seasonal variations in the distribution of rotavirus was not possible to establish. Additionally, other causes of diarrhoea among the respondents were not looked at in this study and rotavirus disease burden from this region is not representative of the burden in the entire country.

### What is known about this topic

- Rotavirus is a leading cause of morbidity and mortality among children under five years with acute gastroenteritis;
- A great diversity of rotavirus strains are co-circulating in the human population throughout the world; the prevalence of rotavirus genotypes varies according to location and time; throughout the world, genotyping and serotyping studies have identified common cocirculating rotavirus types and G1P[8], G2P[4], G3P[8] and G4P[8] are the predominant strains;
- Use of vaccination was found the best way to prevent severe rotavirus infections.

### What this study adds

- For the first time, this study has established the prevalence of rotavirus infection in North Eastern Kenya: no other studies were conducted on the same subject prior to this study;
- This study has also established that the circulating genotypes of rotavirus in this part of the country are similar to the genotypes found in other parts of the country, despite variation in genotype distribution overtime from location to location;
- This study has also established that the circulating rotavirus genotypes in this part of the country are the vaccine preventable types and thus strongly recommended the introduction of rotavirus vaccines into the National immunization program to prevent severe rotavirus infection among children under five years.

## Competing interests

The authors declare no competing interest.
